# TRANSHIATAL ESOPHAGECTOMY IS NOT ASSOCIATED WITH POOR QUALITY LYMPHADENECTOMY

**DOI:** 10.1590/0102-672020190001e1475

**Published:** 2019-12-20

**Authors:** Flávio Roberto TAKEDA, Francisco TUSTUMI, Bruna de Camargo NIGRO, Rubens Antonio Aissar SALLUM, Ulysses RIBEIRO-JUNIOR, Ivan CECCONELLO

**Affiliations:** 1 Gastroenterology Department, University of São Paulo, São Paulo, SP, Brazil

**Keywords:** Esophageal neoplasms, Neoadjuvant therapy, Esophagectomy, Terapia neoadjuvante, Esofagectomia, Neoplasias esofágicas

## Abstract

**Background:**

Esophageal cancer neoadjuvant therapy followed by surgery increases the likelihood of treatment success.

**Aim:**

To evaluate variables that can influence the number of retrieved lymph nodes, the number of retrieved metastatic lymph nodes and lymphnodal recurrence in esophagectomy after neoadjuvant chemoradiotherapy.

**Methods:**

Patients of a single institute were evaluated after completion of trimodal therapy. Univariate and multivariate analyses were performed to evaluate variables that can influence in the number of retrieved lymph nodes and retrieved metastatic lymph nodes.

**Results:**

One hundred and forty-nine patients were included. Thoracoscopy access was considered an independent factor for the number of lymph nodes retrieved, but was neither related to the number of positive lymph nodes retrieved nor to lymphnodal recurrence. Pathological complete response on the primary tumor and male were independent variables associated with the number of positive lymph node retrieved. Pathological complete response on the primary tumor site did not statistically influence the likelihood of a lower number of lymph nodes retrieved.

**Conclusion:**

Patients submitted to esophagectomy after neoadjuvant chemoradiotherapy, thoracoscopic access is more accurate for pathological staging, even in a complete pathological response. With a proper patient selection, transhiatal surgery may preserve the quality of lymphadenectomy of the positive lymph nodes.

## RESUMO

**Racional:**

No câncer esofágico a terapia neoadjuvante seguida de procedimento cirúrgico aumenta a probabilidade de sucesso do tratamento.

**Objetivo:**

Avaliar variáveis que podem influenciar o número de linfonodos recuperados, o número de linfonodos metastáticos recuperados e a recorrência linfonodal na esofagectomia após quimiorradioterapia neoadjuvante.

**Métodos:**

Os pacientes incluídos foram aqueles que terminaram terapia trimodal. Análises univariadas e multivariadas foram realizadas para avaliar as variáveis que pudessem influenciar no número de linfonodos recuperados e nos metastáticos recuperados.

**Resultados:**

Cento e quarenta e nove pacientes foram incluídos. O acesso por toracoscopia foi considerado fator independente para o número de linfonodos recuperados, mas não teve relação com o número de linfonodos positivos recuperados, nem com recorrência linfonodal. Resposta patológica completa no tumor primário e homens foram variáveis independentes associadas ao número de linfonodos positivos recuperados. A resposta patológica completa do tumor primário não acarretou em número menor de linfonodos recuperados.

**Conclusão:**

Em pacientes submetidos à esofagectomia após quimiorradioterapia neoadjuvante o acesso toracoscópico é mais preciso para estadiamento patológico, mesmo em resposta patológica completa. Com seleção adequada a esofagectomia trans-hiatal pode preservar a qualidade da linfadenectomia dos linfonodos positivos.

## INTRODUCTION

Esophageal cancer is a growing health issue expected to increase in incidence over the next years due to the upsurge of gastroesophageal reflux disease and obesity^4^ ^,^ ^15^. It is still a challenging disease with a poor prognosis despite the advances in chemoradiotherapy^2^ and all the progress made in the last years in minimally invasive surgery and postoperative management^19^. 

The prognosis of this aggressive neoplasm depends mainly on the local invasion extension, hematogenous metastasis, and lymphatic spread^20^. Surgical resection is the primary treatment for localized esophageal carcinoma, and a properly lymphadenectomy is crucial for long-term survival^12^. Both the number of lymph nodes retrieved during esophagectomy and the metastatic lymph correlated with survival rates^1^ ^,^ ^21^. Also, lymph node status is crucial for neoplasm staging and, thus, for establishing prognosis and treatment strategies^7^ ^,^ ^11^ ^,^ ^23^. 

On the other hand, good quality lymphadenectomy may be challenging after chemoradiation, once it leads to the formation of fibrosis inside the radiation field^8^ ^,^ ^9^ ^,^ ^18^. Also, systemic neoadjuvant chemotherapy may modify the number and the distribution of mediastinal lymph nodes and also affect metastatic lymph nodes^9^. At the same time, neoadjuvant chemoradiation therapy is the standard treatment for most localized esophageal neoplasms, once it can improve long-term survival by controlling of locoregional disease, tumor downstaging before surgery, and targeting micrometastases, decreasing the risk of distant metastasis^14^ ^,^ ^22^. 

This retrospective cohort study aims to evaluate the variables that can influence the quality of lymphadenectomy in esophagectomy after neoadjuvant chemoradiotherapy using platinum- and taxane-based regimen. For lymphadenectomy quality assessment, this study evaluated the number of lymph nodes retrieved (LNr), the number of positive lymph nodes retrieved (PLNr), and lymphnodal recurrence rate.

## METHODS

This study was approved by institutional ethics committee with number 3.315.331

### Study design

This is a retrospective cohort in which patients of a single institute with completion of neoadjuvant chemoradiotherapy using platinum- and taxane-based regimens, followed by curative intent esophagectomy, were selected. Radiation dose ranged from 41.4 to 50.4 cGy. A transthoracic approach with two-field lymph-node dissection was performed for tumors extending proximally to the tracheal bifurcation. A transhiatal resection was preferred for tumors involving the esophagogastric junction. Patients were recruited from 2009 to 2019, and were staged with endoscopy, CT-scan, and PET-Scan before neoadjuvant therapy and classified according to the 8^th^ edition of UICC staging^16^. Patients were followed with clinical exams, endoscopies, and CT-scans. The endpoints evaluated were the number of lymph nodes retrieved (LNr), the number of positive lymph nodes retrieved (PLNr), and lymphnodal recurrence rate. 

### Statistical analysis

Chi-square test or likelihood-ratio test were used for each outcome for absolute and relative variables. Kruskal-Wallis and ANOVA tests were used to assess for significant differences on a continuous dependent variable by a categorical independent variable. Multivariate Cox proportional hazard analysis was performed to determine independent risk factors for the outcomes. Only variables that were significant on univariate analysis were included as covariates in the multivariable analyses. Data were assessed with IBM-SPSS software version 20.0, and a significance level of 0.05 was adopted.

## RESULTS

Were enrolled 149 consecutive patients between 2009 and 2019 that have undergone neoadjuvant chemoradiotherapy using platinum- and taxane-based regimen followed by curative intent esophagectomy. The mean age was 61.5 years (SD±8.1), with male predominance (76%). There were 101 squamous cell carcinoma (SCC) and 48 esophageal adenocarcinoma patients. The mean follow-up was 31.3 months (SD±22).

The median number of lymph nodes retrieved (LNr) was 19. Patients were grouped as up to 19 and more than 19 LN for univariate analysis (Table 1). Surgical access (p<0.001) and age (p=0.003) influenced the number of LNr in esophagectomy. Pathological complete response (pCR) on the primary tumor site did not statistically influence the likelihood of a lower number of LNr (OR 0.81; 95%CI: 0.41-1.56; p=0.536). When analyzing each surgical access separately (transhiatal or thoracoscopy), pCR was still not statistically associated with the number of LNr (Table 2). In multiple regression analysis, only thoracoscopy access was considered an independent factor (OR 6.4; 95%CI: 2.18-18.75; p=0.001).

**TABLE 1:** Univariate analysis for number of lymph node retrieved

Variable	Lymph node retrieved		OR	CI (95%)		p
	≤19 (n=76)	>19 (n=73)		Lower	Upper	
Gender, n (%)						0.807
Male	57 (50.4)	56 (49.6)	1.00			
Female	19 (52.8)	17 (47.2)	0.91	0.43	1.93	
Histology, n (%)						0.856
SCC	51 (50.5)	50 (49.5)	1.00			
Adenocarcinoma	25 (52.1)	23 (47.9)	0.94	0.47	1.87	
Surgical access, n (%)						<0.001
Transhiatal	27 (84.4)	5 (15.6)	1.00			
Thoracoscopy	49 (41.9)	68 (58.1)	7.49	2.70	20.83	
cStage, n (%)						0.174
I/II	23 (60.5)	15 (39.5)	1.00			
III/IV	53 (47.7)	58 (52.3)	1.68	0.79	3.55	
Grade of cellular differentiation, n (%)						0.370
Well	9 (69.2)	4 (30.8)	1.00			
Moderately	47 (48.5)	50 (51.5)	2.39	0.69	8.30	
Poorly	18 (50)	18 (50)	2.25	0.59	8.65	
pCR, n (%)						0.536
No	42 (48.8)	44 (51.2)	1.00			
Yes	34 (54)	29 (46)	0.81	0.41	1.56	
Age (years)						0.003**
median (min; max)	63 (42;79)	60 (37;76)	0.941	0.904	0.981	
Interval CRT-surgery (days)						0.169**
median (min; max)	96 (38;288)	94 (31;293)	0.995	0.987	1.002	

Chi-squared test **Mann-Whitney

**TABLE 2:** There was no association of lymph node retrieved and pathological complete response (pCR)

		Lymph node retrieved		Total	p
Thoracoscopy		≤19	>19		
pCR (%)	No	26 (39.4)	40 (60.6)	66	0.535
	Yes	23 (45.1)	28 (54.9)	51	
Total (%)		49 (41.9)	68 (58.1)	117	
Transhiatal		≤19	>19		
pCR (%)	No	16 (80)	4 (20)	20	0.626
	Yes	11 (91.7)	1 (8.3)	12	
Total (%)		27 (84.4)	5 (15.6)	32	

Fisher’s exact test Fisher’s exact test

The number of positive lymph nodes retrieved (PLNr) was associated with gender (p=0.044), adenocarcinoma histology (p<0.001), and pCR on the primary tumor site (p<0.001) on univariate analysis (Table 3). In the multiple regression analysis, pCR on the primary tumor (OR: 0.1; 95%CI: 0.04-0.25; p<0.001) and gender (male patients were more likely to have PLNr than women (OR 2.69; 95%CI: 1.03-7.04; p=0.044) were independent variables associated with the number of PLNr. Surgical access was not related to PLNr.

**TABLE 3:** Univariate analysis for metastatic lymph nodes

Variable	Metastatic lymph node (%)		OR	CI (95%)		p
	No (n=86)	Yes (n=63)		Lower	Upper	
Gender, n (%)						0.044
Male	67 (59.3)	46 (40.7)	1.00			
Female	28 (77.8)	8 (22.2)	0.42	0.17	0.99	
Histology, n (%)						<0.001
SCC	74 (73.3)	27 (26.7)	1.00			
Adenocarcinoma	21 (43.8)	27 (56.3)	3.52	1.71	7.24	
Surgical access, n (%)						0.867
Transhiatal	20 (62.5)	12 (37.5)	1.00			
Thoracoscopy	75 (64.1)	42 (35.9)	0.93	0.42	2.10	
cStage, n (%)						0.140
I/II	28 (73.7)	10 (26.3)	1.00			
III/IV	67 (60.4)	44 (39.6)	1.84	0.81	4.16	
Grade of cellular differentiation, n (%)						0.323#
Well	8 (61.5)	5 (38.5)	1.00			
Moderately	65 (67)	32 (33)	0.79	0.24	2.60	
Poorly	19 (52.8)	17 (47.2)	1.43	0.39	5.23	
pCR, n (%)						<0.001
No	39 (45.3)	47 (54.7)	1.00			
Yes	56 (88.9)	7 (11.1)	0.10	0.04	0.25	
Age (years)						0.298**
median (min; max)	63 (41;79)	61 (37;78)	0.979	0.941	1.018	
Interval CRT-surgery (days)						0.912**
median (min; max)	94 (36;293)	98 (31;244)	0.999	0.991	1.006	

Chi-squared test **Mann-Whitney test

Concerning lymphnodal recurrence, only pCR of the primary tumor was statistically significant (OR: 0.327; 95%CI: 0.08-0.99; p=0.038, Table 4). The surgical access was not related to lymphnodal recurrence.

**TABLE 4:** Univariate analysis for lymph nodal recurrence

Variable	Lymph nodal recurrence		HR	CI 95%		p
	No (n=126)	Yes (n=23)		Lower	Upper	
Gender, n (%)						
Male	97 (85.8)	16 (14.2)	1,00			
Female	29 (80.6)	7 (19.4)	1.32	0.554	3.15	0.53
Histology, n (%)						
Adenocarcinoma	41 (85.4)	7 (14.6)	1,00			
SCC	85 (84.2)	16 (15.8)	1.01	0.418	2.45	0.98
Surgical access, n (%)						
Transhiatal	30 (93.8)	2 (6.3)	1,00			
Thoracoscopy	96 (82.1)	21 (17.9)	2.91	0.681	12.4	0.15
cStage, n (%)						
I/II	32 (84.2)	6 (15.8)	1,00			
III/IV	94 (84.7)	17 (15.3)	0.981	0.389	2.47	0.97
pCR	58 (92)	5 (8)	0.327	0.08	0. 99	0.038
Grade of cellular differentiation, n (%)						
I	10 (76.9)	3 (23.1)	1,00			
II	82 (84.5)	15 (15.5)	0.863	0.368	2.02	0.73
III	31 (86.1)	5 (13.9)	0.945	0.351	2.54	0.91
Age (yr)			0.965	0.921	1.01	0.13
Mean±SD	61.4±8.6	58.6±8.4				
Median (min; max)	62.5 (41; 79)	60 (37; 71)				
Interval CRT-Surgery (days)			1,00	0.996	1.01	0.49
Mean±SD	102.1±47.9	111.1±36.5				
Median (min; max)	94 (31; 293)	98 (59; 177)				

Chi-squared test

## DISCUSSION

This cohort comprising 149 patients submitted to neoadjuvant chemoradiotherapy using platinum- and taxane-based regimen followed by curative intent esophagectomy showed that thoracoscopy access was more likely to achieve lymph node retrieval than transhiatal access. On the other hand, surgical access did not differ in the number of positive lymph nodes retrieved, and also did not influence the risk for lymphnodal recurrence.

The transthoracic access provides a better pathological staging since it showed a higher number of LNr. However, the number of LNr is not the same for the good quality lymphadenectomy. In fact, with proper patient selection, transhiatal access with infracarinal lymphadenectomy may be enough to retrieve the nodal stations with a higher risk for positivity, avoiding lymphnodal recurrence. In this scenario, transhiatal approach turns to be a good option, since it is associated with reduced perioperative morbidity, a shorter hospital stay, and decreased in-hospital mortality rates^6^ ^,^ ^13^, mainly in the cases of laparoscopic transhiatal esophagectomy^3^.

Neither complete pathological response nor histological type was associated with the number of retrieved lymph nodes. Therefore, the number of LNr should be taken into consideration as one of the parameters when monitoring the quality of esophagectomy for cancer after neoadjuvant therapy, regardless of pathological response to neoadjuvant therapy or histological type. This finding differs from rectal cancer, in which pCR after chemoradiotherapy is associated with a lower number of lymph nodes harvested^5^. Squamous cell carcinoma showed a higher likelihood of a lower number of positive lymph nodes retrieved, but this is probably due to the higher response to neoadjuvant therapy.

Gender was associated with a higher likelihood of PLNr. While the mechanism underlying the association of lymph nodes metastasis and gender, epidemiological studies have reported a potential association between gender hormones and certain neoplasms, such as colorectal and thyroid cancers^10^ ^,^ ^17^.

This study has several limitations. It was a retrospective, nonrandomized, single-institution; the number of patients, especially with adenocarcinoma, was relatively small; and different neoadjuvant chemotherapy other than platinum- and taxane-based regimen, were not evaluated. Further studies with large controlled trials, comparing transhiatal and transthoracic access patients are required.

## CONCLUSION

Thoracoscopic access is more accurate for pathological staging in patients submitted to esophagectomy after neoadjuvant chemoradiotherapy using platinum- and taxane-based regimen, even for a complete pathological response. With proper selection, transhiatal surgery may preserve the quality of lymph node excision of the positive lymph nodes.
